# Emerging Importance of Survivin in Stem Cells and Cancer: the Development of New Cancer Therapeutics

**DOI:** 10.1007/s12015-020-09995-4

**Published:** 2020-07-20

**Authors:** Neerada Meenakshi Warrier, Prasoon Agarwal, Praveen Kumar

**Affiliations:** 1 Department of Biotechnology, Manipal Institute of Technology, Manipal Academy of Higher Education, Manipal, 576104 KA India; 2 KTH Royal Institute of Technology, School of Electrical Engineering and Computer Science, Stockholm, Sweden; 3 Science for Life Laboratory, Solna, Sweden

**Keywords:** Survivin, Cancer stem cells, Cancer signaling, Anticancer therapy, Inhibitors

## Abstract

Survivin is one of the rare proteins that is differentially expressed in normal and cancer cells and is directly or indirectly involved in numerous pathways required for tumor maintenance. It is expressed in almost all cancers and its expression has been detected at early stages of cancer. These traits make survivin an exceptionally attractive target for cancer therapeutics. Even with these promising features to be an oncotherapeutic target, there has been limited success in the clinical trials targeting survivin. Only recently it has emerged that survivin was not being specifically targeted which could have resulted in the negative clinical outcome. Also, focus of research has now shifted from survivin expression in the overall heterogeneous tumor cell populations to survivin expression in cancer stem cells as these cells have proved to be the major drivers of tumors. Therefore, in this review we have analyzed the expression of survivin in normal and cancer cells with a particular focus on its expression in cancer stem cell compartment. We have discussed the major signaling pathways involved in regulation of survivin. We have explored the current development status of various types of interventions for inhibition of survivin. Furthermore, we have discussed the challenges involving the development of potent and specific survivin inhibitors for cancer therapeutics. Finally we have given insights for some of the promising future anticancer treatments.

## Introduction

Survivin, an evolutionarily conserved inhibitor of apoptosis protein (IAP) is a multi-tasking extraordinaire, with differential expression patterns in normal and cancer cells. The copious information available on this protein, since its emergence has been vibrantly captivating the attention of both cell biologists and oncologists. This protein, central to various cellular signaling pathways and instrumental in cell proliferation and programmed cell death, has transpired as a cardinal onco-therapeutic target in the last two decades [1–3]. This protein performs important roles in cell cycle, apoptosis, angiogenesis and cancer formation and progression. It is found in different subcellular fractions and the major pools in cancer cells are in nucleus, cytoplasm, mitochondria and extracellular space [4]. Nuclear survivin regulates cell division while cytoplasmic expression is associated with cytoprotection for tumors. The balance between these two pools of the protein is indicative of ‘active survivin’ and the relative intracellular expression level quantification helps in analyzing the prognostic significance of the marker [5]. Mitochondrial survivin is associated with tumor growth and apoptotic resistance while extracellular survivin can re-enter tumor cells and increase proliferation, apoptotic resistance and invasion. The extracellular survivin is localized on exosomes secreted by cancer cells. This pool plays a role in cell-cell communication [2, 6].

## Structure and Functions of Survivin

The 142 amino acid long survivin, encoded by *BIRC5* (Baculoviral IAP repeat (BIR) - containing 5) gene is the smallest of the eight IAPs in human genome and is located at the telomeric end of chromosome 17 (17q25). It consists of a 70 amino acid long N-terminal BIR domain associated with apoptotic function, a carboxy terminal α-helix region that binds with BIR domain involved in mitotic function and a dimer binding domain [7, 8]. Studies have shown an increased expression of the protein in the G2/M phase of cell cycle, thus aiding in cell division and mitosis. This protein attaches to mitotic spindle through the α-helix, during the short gap between metaphase and anaphase, and in association with tubulin and constituent elements of the mitotic apparatus regulates microtubule dynamics and nucleation [9, 10]. Another pool of survivin localizes to the kinetochores and with the help of cytokinesis regulators, Aurora B kinase and inner centromere protein (INCEP), helps in chromosome segregation and cytokinesis [11, 12]. Thus the protein is a member of the chromosome passenger complex (CPC) which has significant role in regulation of chromosome-microtubule attachment, bipolar spindle formation, spindle assembly checkpoint and cytokinesis at cell division [13].

**Fig. 1:**
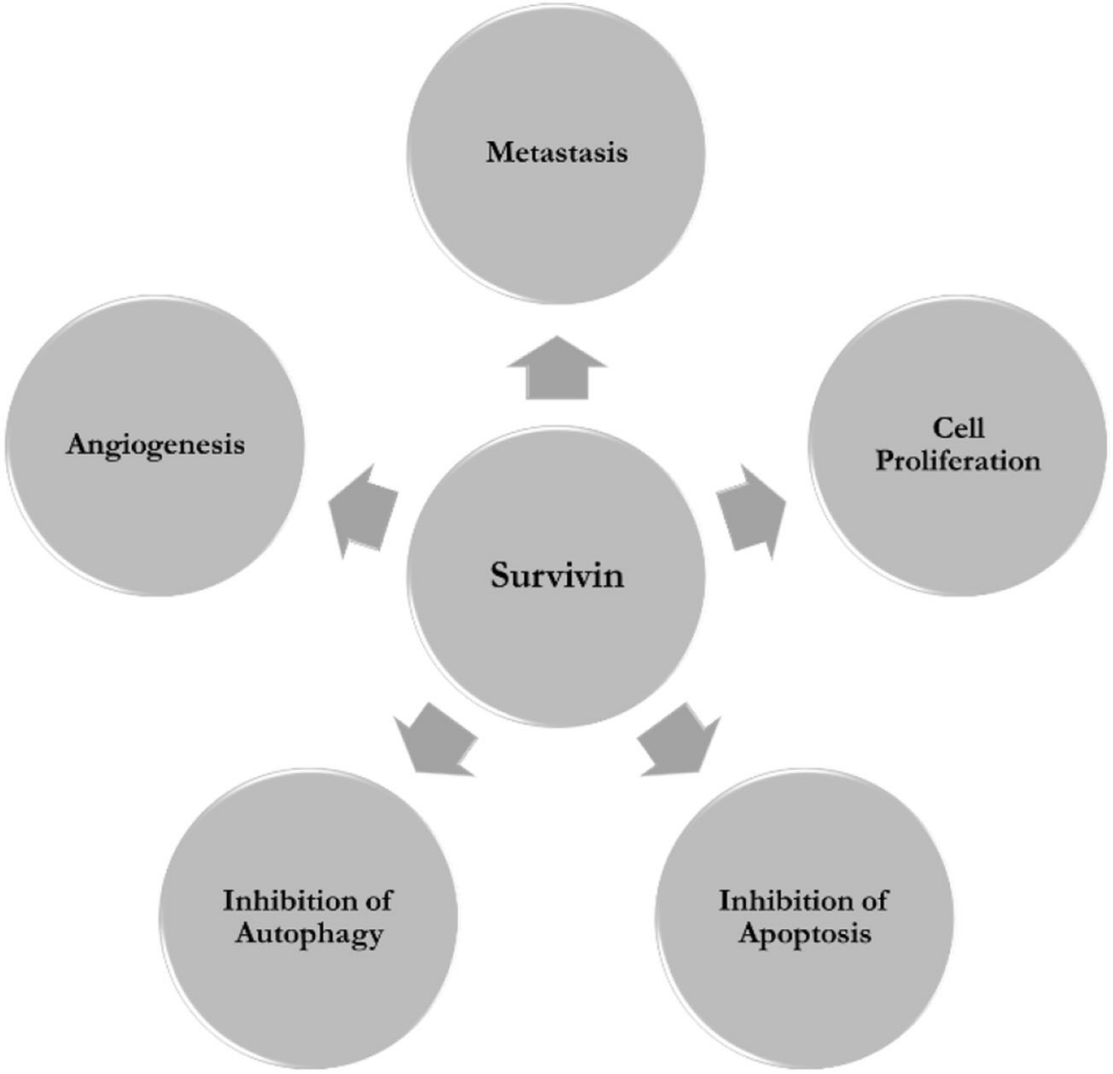
Survivin as a nodal protein

Survivin inhibits both apoptotic and autophagic cell death [14]. It inhibits extrinsic and intrinsic apoptotic pathways by both caspase independent and dependent mechanisms. While other IAPs bind to and directly degrade the active caspases 3, 7 and 9, survivin binds to and suppresses pro-caspase 9 with the help of hepatitis B X-interacting protein (HBXIP) cofactor, thus inhibiting death receptor signaling pathway [15, 16]. The interaction of mitochondrial survivin with X linked IAP (XIAP), resulting in the formation of an IAP-IAP complex can also suppress caspase 9 [17]. They also sequester and inactivate second mitochondria-derived activator of caspase/direct inhibitor of apoptosis-binding protein with low pI (Smac/DIABLO), that otherwise act as an IAP antagonist preventing the binding of XIAP to survivin thus releasing caspase 9 [8]. Even though increase in survivin expression has been reported in relation to inhibition of autophagy the exact mechanism and interacting proteins have not been determined yet [14]. The role of survivin in enhancing vascular endothelial growth factor (VEGF) transcription, synthesis and release and promoting vasculogenic mimicry in hypoxic conditions, substantiates its involvement in angiogenesis and subsequent upregulation in angiogenically stimulated cells [18]. Survivin also binds to c-Src (cellular sarcoma) establishing a bidirectional relationship that adversely affects focal adhesion dynamics and integrity of F-actin organization and disrupting cell-cell and cell-matrix interactions [19]. Moreover survivin is also involved in DNA damage repair, tissue response to injury and immune response [20]. Figure. 1 illustrates the major roles of survivin.

The protein has 3 introns and 4 exons and in addition to survivin wild type (WT) produces various isoforms, survivin 2b (S-2b), survivin 2a (S-2a), survivin 3b (S-3b), Survivin ΔEx3 (S-ΔEx3), survivin 2α (S-2α), survivin 3α (S-3α), survivin-3γ (S-3γ), survivin-3γV (S-3γV), survivin 2b + 32 (S-2b + 32) and survivin image (SI). Each of these variants has different intracellular localization, expression patterns and can form heterodimers with WT. Some splice variants with unknown biological functions, including survivin-ΔptEx1/2, survivin-ΔptEx1/2G/T, survivin-ΔptEx2/3 and survivin-ΔptEx2, have also been reported. Variants S-ΔEx3, S-3b, S-2α, S-3α, S-3γ and S-3γV are all antiapoptotic while S-2a and S-2b are pro-apoptotic due to the missing and truncated BIR domain. S-ΔEx3 is associated with cell mortality and often with angiogenesis [21–23]. S-3b is cytoprotective, associated with cell cycle regulation and helps in evading immune response[24].

## Survivin in Non-malignant Non-Stem Cells

Even though a few human adult tissues like primitive hematopoietic cells, adult peripheral blood T cells, polymorphonuclear neutrophils, erythroid cells, megakaryocyte, vascular endothelial cells, cells of colonic and gastrointestinal mucosa, placenta, testes, ovary, neurons, melanocytes etc. express survivin, the presence is almost negligible in most terminally differentiated tissues [25–29]. The more mature the tissue the lesser the expression of the protein [20]. They have roles in vascular remodeling, angiogenic regulation, cell cycle regulation and proliferation, maintenance of normal adult hematopoiesis, erythropoiesis, megakaryocyte maturation, hepatocyte proliferation, apoptosis inhibition in neural development, self-renewal and differentiation of cryptic stem cells, spermatogenesis and oogenesis [27–29].

Even though most normal tissues do not express the splice variants, some do express minimal amounts of certain isoforms. S-ΔEx3 expression is observed in vascular endothelial cells. S-ΔEx3 is the only isoform expressed in adult brain cerebrum while fetal brain tissues showed the expression of WT and S-2b as well. Colon and soft sarcoma tissues did show trace amount of these variants in certain cases [23]. Minimal expression of S-2α has also been observed in normal brain cerebellum and breast tissues [30]. Expression of other splice variants are unknown till date.

## Survivin in Stem Cells

Increased survivin expression has been reported in early embryonic stem cells (ES) wherein it reduces during fetal development and also some adult stem cells. Adida et al. reported survivin expression in apoptosis regulated human fetal tissues including thymic medulla, stem cell layer of stratified epithelia of skin, gastrointestinal tract and endocrine pancreas, preserving the viability of stem cells in a developmentally regulated fashion thus helping in tissue homeostasis and differentiation [31]. The analysis of the protein expression in umbilical cord blood cells and adult bone marrow CD34^+^ cells showed that the protein is hematopoietic cytokine regulated and hematopoietic growth factor dependent. The protein expression is observed in all stages of cell cycle and showed an inverse correlation with apoptosis in caspase dependent manner [32]. Survivin upregulation is a mandatory process for entry to cell cycle, self-renewal and maintenance in normal cord blood CD34^+^ cells and hematopoietic stem and progenitor cells (HSPCs) [33, 34]. A work by Filion et al. showed that in human embryonic stem cells (HES) subjected to ionizing radiation the expression of this IAP along with checkpoint kinase 2 (Chk2) helps to recuperate DNA damage without the dependency on p21 to support cell cycle arrest [35]. Higher survivin levels correlate to maintenance of the state of pluripotency in HES and induced pluripotent stem cells (iPSCs). The protein is involved in the regulation of cell proliferation and signaling pathways in stem cells [20]. A study conducted to understand the role of survivin in pluripotency showed a significantly higher expression of the protein in human embryonic stem cells (almost 9 fold) in comparison to that in differentiated cell types [36]. The IAP is involved in controlling basal and growth factor dependent survival, proliferation, differentiation and migration of mouse and human mesenchymal stem cells (MSCs). The expression is observed in all stages of interphase with exceptionally high expression in G2/M phase. Increased survivin expression protects MSCs that form HSCs supportive niche depleted post irradiation exposure [37]. The protein has been linked to survival in these cells subjected to stress induced by heat shock, UV or etoposide as well [38]. CD44^+^/CD105^+^ human amniotic fluid MSCs, that proliferate rapidly and are ideal for stem cell transplantation, also express high levels of survivin [39]. The involvement of hematopoietic adipocyte-derived stem cells (ASCs) in apoptotic resistance displayed by adipocyte tissue in obese subjects was correlated to upregulated survivin expression [40]. Keratinocyte Stem Cells (KSCs) show high expression of survivin, that plays a prominent role in preventing abnormal mitosis in these cells [41, 42]. The suitability of the IAP as a marker for KSCs, which helps in maintaining skin homeostasis has been explored [43]. The role of survivin in the regulation of cell physiology has also been demonstrated in neuronal stem cells and intestinal stem cells [44, 45].

HES expresses S-ΔEx3, S-2b, S-2α, S-3b along with WT in different subcellular locations. The expression is consistently high in HES in comparison to differentiated cell types like MSCs. S-ΔEx3 shows the highest expression levels followed by S-2b. The other two are expressed in minimal quantities [36]. S-3γ and S-3γV isoforms with expression higher than WT are responsible for imparting growth factor independent growth in HSPCs [46]. KSCs as well express S-ΔEx3 and S-2b at higher levels than S-2α, S-3b [42].

## Survivin in Cancer

The overexpression of survivin in majority of carcinomas as well as in the early embryonic stages combined with its relative absence in most normal adult tissues, classifies it as a classic oncofetal gene [47]. A deregulation in the usual expression pattern, resulting in its overexpression throughout the cell cycle is detected in transformed cells and is found to be mediated by oncogenes or tumor suppressor genes, explaining the selective expression of survivin gene in cancers [48, 49]. The survivin pool in mitochondria was found to be involved in cancer [50]. Molecular profiling studies correlated increased survivin expression to aggressive and more invasive tumor behavior, reduced response to drugs, poor prognosis, abbreviated survival and enhanced recurrence [1, 13].

A clear majority of malignancies, including blood, breast, colon, ovarian, lung, liver, uterus, glioblastoma, astrocytoma, meningioma, bladder, prostate, gastrointestinal, non-melanoma skin cancer, melanoma, soft tissue sarcoma and with viral induced neoplasms show overexpression of survivin at different stages in tumor development [51–54]. Overexpression of the protein also acts as a key factor for radio and chemoresistance in various cancers.

Presence of survivin can be detected from body fluids of cancer patients or using survivin antibodies circulating in blood thus acting as an effective diagnostic marker [4]. Detection of survivin from urine of bladder cancer patients, proved to be a simple, yet sensitive diagnostic method for determining new or recurrent bladder cancer [55]. A study to identify survivin positive circulating breast cancer cells in peripheral blood, suggested its presence in more than half of breast cancer patients, while being absent in healthy controls. Similar studies involving survivin levels in serum also showed survivin to be a sensitive diagnostic marker for tumors [56]. Increased expression of the protein in hematological malignancies have suggested the possibility of using this as a common biomarker for detecting these types of diseases [57].

Isoform S-ΔEx3 has been expressed in a wide variety of cancers and is often found in association with malignant cancers and is often reported to be indicative of tumor grade and invasiveness. Studies conducted on S-2b report some contradictory results on the expression of the isoform. Some reports suggested the expression to be positively correlated with adverse clinical outcome while a few suggested an inverse correlation. In cancers the expression was reported to be more in benign or early stage tumors. Certain cases of breast cancer reported S-2α, S-3α and S-3b expression along with WT, S-ΔEx3 and S-2b. S-2α expression has also been observed in medulloblastoma. The expression of ΔptEx2/3 and ΔptEx1/2 are observed in acute myeloid leukemia (AML). S-3α and S-ΔEx3 have potentiality as diagnostic markers for breast and papillary thyroid cancers respectively [23, 30, 58].

## Survivin in Cancer Stem Cells

Cancer stem cells (CSCs) within cancers self-renew, differentiate, accumulate mutations and extensively proliferate, regenerating tumors consisting of both tumorigenic and non-tumorigenic cells, thus sustaining cancer [59]. These cells disregard the normal rules of cell division and the pathways that regulate them, resist the intrinsic and extrinsic apoptotic pathways and improve metastasis. They are believed to be responsible for the relapse and recurrence of cancers and resistance to therapies [60].

To study the mechanisms underlying teratoma formation in HES Blum et al. analyzed the transcriptome of undifferentiated HES cells, teratoma and mature embryoid bodies that showed the upregulation of survivin in HES and teratoma. They reported it to be the strongest candidate gene and also suggested that the steady expression of survivin leads to teratoma formation by increasing apoptotic resistance [47].

Survivin upregulation has been reported to be responsible for the development of hematological malignancies and tumor formation. There is an increased risk of hematologic malignancies in transgenic mice overexpressing survivin due to increased protection from cell death [61]. Increased expression of the protein has been reported in CD34^+^38^−^ AML stem/progenitor cells in comparison to bulk blasts and CD34^−^ AML cells. The expression levels were higher in bone marrow samples in comparison to peripheral blood and the overexpression is often linked to expression of other proteins participating in proliferation and survival [62]. Fukuda *et.al.* reported the regulation of 137 gene by survivin in leukaemia stem cells (LSCs) of which 124 genes were exclusively expressed in LSCs and not in normal HSCs. Genes responsible for wide range of biological functions are regulated by the IAP and these are connected through epidermal growth factor receptor (EGFR) signalling network, suggesting the therapeutic potential of the IAP [63].

Upregulation has been observed in well characterized patient derived glioblastoma stem cell (GSC) lines on comparing the mRNA expression between GSC and non-GSC. The elevated expression of the IAP was observed in recurrent glioblastomas suggesting the possibility of the protein to contribute to therapy resistance in GSCs and its prognostic value in predicting postsurgical survival [64]. Similarly the analysis of gene mRNA expression of CD133^+^ GSCs, reported higher expression in comparison to CD133^−^ cells. This indicates their role in modulation of cell cycle progression, cell division, signaling pathways and increased chemoresistance [65].

Flowcytometric analysis of colorectal CSCs (CR-CSC) revealed differential expression of survivin under different growth conditions. Sphere cells showed lower survivin levels than differentiated cells in adherence conditions, implying the slow growth rate of CSCs [66]. Li et al. observed elevated levels of survivin in CD133^+^ CR-CSCs than in small interfering ribonucleic acid (siRNA)-induced CD133^−^ cells [67]. Similar elevations in levels have been reported in studies conducted in HCT-116 cell line, used as a model for colorectal cancer initiating cells with stem-like cells properties [68]. Transcriptome analysis of CD133^+^ CR-CSCs showed survivin regulated by Interleukin (IL)-4 to have prognostic value in tumor recurrence and patient survival [69]**.** Coexpression of Survivin and CD44^+^, in combination with epithelial mesenchymal transition (EMT) markers were indicative of recurrence and aggressive tumor behavior in rectal cancer patients subjected to preoperative radio and chemotherapy [70].

The significance of the expression of this protein in relation to EMT and metastasis has been studied using quiescent breast CSCs in vitro. Increased expression observed in the intermediate pre-metastatic phase helps in inhibiting apoptosis and surviving, under unfavorable and noxious conditions. After the cells attain mesenchymal phenotype and get adapted to the environment, the expression levels showed a significant decrease, thus giving sufficient evidence for the involvement of survivin in tumor development and progression and its potential as a good CSC marker [71].

Studies conducted by Yie S.M et al. reported the presence of survivin positive circulating cancer cells (CCCs) in peripheral blood samples of about 50% of patients with breast, gastric, lung, colorectal and esophageal squamous cell carcinomas, along with their association with various clinicopathological parameters like degree of tumor infiltration, nodal status and disease stages. These studies suggested the applicability of the protein in predicting metastasis and relapse, thus the possibility of using it as an ideal marker for migrating CSCs [56, 72, 73].

Increased survivin expression in association with increase in stemness has also been reported in prostate CSCs. Survivin was co-expressed with one of this transcriptional regulator Runt-related transcription factor 2 (Runx2) and both showed positive correlation with tumor growth in these CSCs [74]. The upregulated expression of stem cell marker, sex determining region Y box 2 (Sox 2) and survivin resulting in inhibited apoptotic pathway and drug resistance, was related to the presence of CSCs in salivary adenoid cystic carcinoma [75]. Radiation resistant CD44^+^/CD24^+^ cervical cancers also expressed survivin along with various other stem cell markers and drug resistant proteins [76].

## Regulation of Survivin Expression in Cancers

Various signaling pathways and associated molecules play a key role in the positive and negative regulation of survivin. At transcriptional level the cell cycle-dependent element/ cell cycle genes homology region (CDE/CHE) elements in the promoter region control cell cycle dependent gene transcriptions. The survivin promoter has multiple sites for binding pro-oncogenic transcription factors including those that may be responsible for its differential expression in normal and cancer tissues, in combination with the multitude of tumorigenic pathways and signaling molecules [77].

Phosphoinositide-3 Kinase (PI3K)/Akt pathway is initiated on the activation of tyrosine kinase receptor that leads to activation of PI3K which then activates Akt molecule instigating cell proliferation and survival. Akt down regulates FOXO (Forkhead box O) subfamily FOXO1 and FOXO3a, that otherwise binds to and mediate acute silencing of *survivin* through the dual specificity phosphatase and tensin homolog (PTEN) [78]. VEGF, angiopoietin-1 (Ang-1), nuclear factor kappa-light-chain-enhancer of activated B cells (NF-ĸB), cyclooxygenase-2 (COX-2), granulocyte-macrophage colony-stimulating factor receptor (GM-CSFR), EGFR, human epidermal growth factor receptor 2 (HER 2) and 3 (HER 3) upregulate survivin through PI3K/Akt mediated pathway [79]. The stimulation of insulin like growth factor-1 (IGF-1) leads to elevated survivin expression with contributions from PI3K/Akt/mTOR (mammalian target of rapamycin) pathways or by inactivating transforming growth factor-β (TGF-β), a negative regulator of the protein [80, 81]. E2F family of transcription factors are involved with cyclin dependent kinases (CDKs) in cell cycle. E2F activators (E2F1, E2F2 and E2F3) bind to survivin promotor and cause transcriptional activation of the gene, mediated by CDE/CHR mechanisms, while E2F4 and E2F5 down regulate survivin [82, 83]. Downregulation of CDK4/Cyclin D complex by survivin allows hepatoma cells to evade apoptotic signaling due to the activation of CDK2/Cyclin E complex and release of p21 **[**84**]**. In HSPCs apoptosis inhibition by survivin is p21 dependent and cell cycle regulation is p21 independent [85]**.**

Wild type-p53 tumor suppressor gene negatively regulates *survivin* at both mRNA and protein levels, thereby affecting p53 mediated apoptotic pathway [86]. p53 mediated survivin repression can be attributed to either modifications in the chromatin within survivin promoter or the elevation of p21 that results in the hypophosphorylation of retinoblastoma protein (pRB) family and transcriptional suppression by E2F family member [87]. It was found that p53 and RB, along with E2F2 that acts downstream of RB pathway, lead to negative regulation of survivin expression [82]. Mouse double minute 2 (MDM2), downstream of Akt, also downregulates p53, leading to survivin upregulation [88, 89]. Survivin along with p53, mitogen-activated protein kinase (MAPK) and c-Myc pathways play a role in the regulation of adult MSCs and thus help in maintaining the homeostasis of ASCs [90].

The binding of Wnt (Wingless/Integrated) to Frizzled receptor leads to adenomatous polyposis coli gene/ glycogen synthase kinase 3beta (GSK-3β) activation leading to build up of β-catenin that activates Wnt/β-catenin pathway. β-catenin/T cell factor transcriptional activator increases survivin levels aiding in inhibition of apoptosis and enhancing proliferation [91, 92]**.** The interaction between survivin and Wnt/β-catenin pathway aids in maintaining the pluripotent state of HES [20]. The improved efficiency of one factor (1F) octamer-binding transcription factor-4 (Oct-4) reprogramming of human neural progenitors to iPSCs can also be attributed to the Survivin/β-catenin interaction [93]. Hypoxia Inducible Factor (HIF-1α) directly binds to survivin promotor and shows a positive correlation specially under hypoxic conditions, while aiding in migration, survival and metastasis [94, 95].

Janus kinase (JAK)/ signal transducer and activator of transcription 3 (Stat3) pathway plays significant roles in cancer progression, migration, apoptosis and immunity. Stat3 oncogene downstream of JAK modulates transcriptional activation and repression of survivin in lymphoma, while the protein directly binds to survivin promotor and inhibit apoptosis in breast cancer cells [96]. The binding of IL-6 or IL-11 to the respective receptors or Bcr-abl activation initiates JAK2 in the cytosol that increases the DNA binding of Stat3 there by activating survivin[97]**.** Bcr-abl upregulates Survivin and the interaction has significant effect on telomerase activity in cells with high c-abl kinase activity [98, 99]. MAPK/extracellular-signal-regulated kinase (ERK) signaling cascade comprising of retrovirus-associated DNA sequences (RAS), rapidly accelerated fibrosarcoma (RAF), Mitogen-activated protein kinase (MEK) and ERK is another pathway leading to survivin gene regulation. Ras-signaling activates MAPK, mTOR and Akt pathways that increase the transcriptional activation of survivin [100]. Ras also plays significant role in the modulation of survivin expression in Ba/F3 hematopoietic cell line helping in their growth factor independent survival and proliferation [101].

Transforming growth factor β (TGF- β) transcriptionally downregulates survivin expression in association with SMADs 2 and 3 and CDE/CHR elements on survivin promoter or through inactivation of RB pocket. Notch signaling pathway induces cell differentiation or maintains cells in an undifferentiated state and aids in stemness in cells. It positively regulates survivin in association with HIF-α and Jagged-1 ligand (RPB-jĸ) in lung cancers [102]. Notch signaling with the help of c-Myc positively regulates survivin thus inducing T lymphocyte differentiation from HSCs [103].

There exists a functional link between survivin and Wee-1 kinase, that prevents the caspase 3 mediated degradation of Wee-1 kinase. This interaction enhances p34Cdc2 phosphorylation, leading to anti-apoptotic activity and improved cell survival in HSCs [104]. Prostaglandin E2 helps in survivin upregulation along with C-X-C motif chemokine receptor 4 (CXCR4) and stromal cell-derived factor (SDF 1), thus enhancing HSC survival and homing [105]. CXCR4/SDF-1 signaling in bone marrow stromal cell niche with crucial involvement of survivin, maintains cell function during hemostasis and promotes hematopoietic recovery [106]. Transcription factor ecotropic virus integration site 1 protein homolog (Evi-1) and its downstream targets Gata2, Pre-B cell leukemia transcription factor 1 (Pbx1) and Spalt Like Transcription Factor 2 (Sall2) are also correlated with survivin expression and associated functions in HSCs [107]**.**

**Fig. 2:**
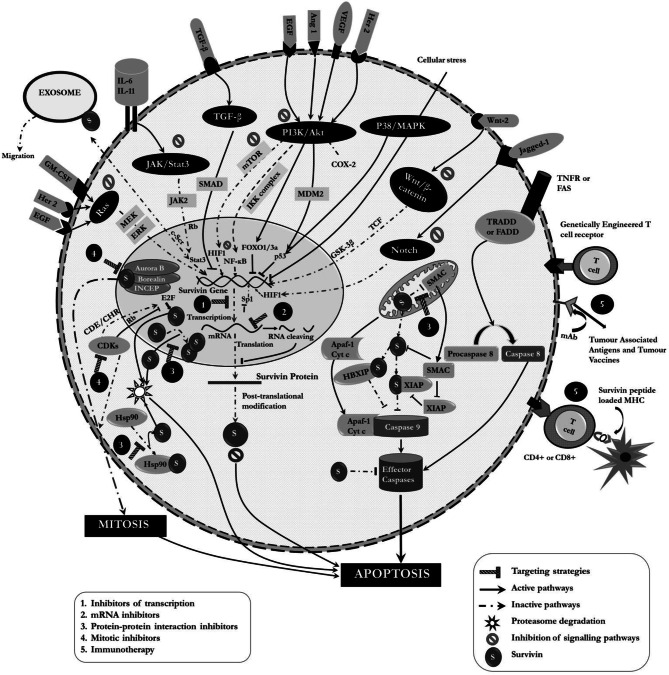
Involvement of survivin in various cellular networks and associated current therapeutic interventions

Survivin has also been implicated in CSC signaling paradigms. It is a major downstream target of the above mentioned pathways along with other genes like Sox-2, Oct-4, c-Myc, Nanog, cyclin D1 etc. [108]. Oct-4 with the help of Stat3 and survivin was significant in the survival of drug resistant CR-CSCs [109]. It is associated with unfavorable prognosis in leukemia stem/progenitor cells and modulation of sonic hedgehog (SHh) and TGF-β pathways, thereby promoting pancreatic and liver CSC growth respectively. It is involved in Stat 3 signaling favoring CSC progression in the niche [110]. Internal tandem duplication-Flt3 tyrosine kinase (ITD-Flt3) upstream of survivin helps in its regulation in a PI3K/Akt dependent manner in AML [111]. Survivin regulation via β-catenin was found to be responsible for radioresistance in PTEN(−) CSC-like cells of nasopharyngeal carcinoma [112]. Kruppel-like factor 5 (KLF5) is also capable of inducing survivin expression by binding to p53 and preventing p53 mediated down regulation of the protein acute lymphoblastic leukemia (ALL), colorectal and ovarian CSCs [77, 113]. Wnt/ β-catenin pathway regulates survivin with accompanied modulation of Cyclin-D1 and c-Myc in liver CSCs [114]. Transient receptor potential cation channel, subfamily M, member 7 (TRPM7) is indirectly involved in survivin regulation in CSCs through Notch and JAK2/Stat3 signaling pathways [115]**.** Figure 2 illustrates the various cellular networks that involve survivin.

## Survivin Targeting Therapeutics

The inhibition of survivin expression can lead to sensitization of tumors to chemical and physical agents as this can inhibit tumor proliferation through a spontaneous increase in the rate of apoptosis. Various small molecule inhibitors, ribozymes, gene editing techniques, antisense oligonucleotides, cancer vaccines etc. have been developed with an aim to achieve the same (Fig. 2, Table 1).

**Table 1:** Survivin therapeutics- Drug targets and mechanism of action in various cancers

Drug	Target	Mechanism of action	Cancers
Inhibitors of Survivin Transcription			
FL118	Survivin Promotor	Multiple mechanisms leading to transcriptional inhibition	Head and Neck, Cervical, Ovarian, Pancreatic, Prostate, Leukaemia, Lung and CSCs [116, 117, 235, 236]
YM155	SP1 binding site of Survivin Promotor	DNA damage and S phase arrest	Leukaemia, Neuroblastoma, Gastric, Pancreatic, Liver, Prostate, NSCLC [119]
Tolfenamic Acid	SP1 and SP3	Loss of DNA binding specificity leading to transcriptional repression	Pancreatic, Ovarian and Ewing Sarcoma [122, 123]
Teremprocol	SP1 and cdc2	Transcriptional Repression	Lung, Colorectal, Breast, Prostate, Liver [124, 125]
Mithramycin A	SP1 and SP3	Loss of DNA binding specificity of SP1	Ewing Sarcoma, LSCs [126, 127, 225]
Protein-Protein interaction inhibitors			
Survivin-Survivin interaction inhibitors			
Abbot 8 LQZ7 LQZ7F	Survivin	Binds to survivin dimer interface	Cervical, Pancreatic [128, 129]
Survivin-Smac interaction inhibitors			
Piperine Withanone UC112 4 g 10f	Survivin	Bind to Survivin at Smac binding region, increases survivin expression	Melanoma, Pancreas, Breast and Colorectal [130–133]
Survivin-Hsp90 interaction inhibitors			
Shepherdin AICAR 17AAG NVP-922	HSP90/ Survivin complex	Disruption of survivin Hsp90 interaction and proteasomal degradation	Prostate, Cervical, Melanoma, Colorectal, Chronic myelogenous Leukaemia [133–140]
Mitotic Inhibitor			
Indinavir	Survivin	Inhibit Aurora B phosphorylation and CPC complex formation	Breast [141]
S12		Microtubule dynamics	Medulloblastoma, Cervical, Osteosarcoma, Breast [141]
LLP3		Affect survivin-Ran stability, and spindle formation	Colorectal, Cervical, GSC [64]
Flavopiridol	CDKs	Hyperphosphorylation at 34th position, mitotic block	Ovarian, Breast, Cervical [142]
Purvalanol A	P34 cdc2		Gastric, Ovarian [143]
NU6140	CDK2	Transcriptional inhibition and caspase activation	Cervical, Ovarian [144]
Cephalochro min	CDK2/ Cyclin -E CDK4/ Cyclin-D1	Mitochondrial disruption and cell cycle arrest	Lung [145]
Inhibitors of Signalling molecules			
Prodigiosin	P53	Caspase activation	Breast, Liver, Colorectal, Leukaemia, CSCs [146]
Omega 3 fatty acids			Colon, Acute Myeloid Leukaemia, CSCs [147, 148, 232]
Nutlin 3a	Integrin-α5β1 MDM2	P53 and mTOR dependent downregulation	Glioblastoma [149, 150]
RG7388	MDM2		Neuroblastoma [149]
Rapamycin	mTOR	Binds to FKBP12	Glioblastoma, Leukaemia, Lymphoma, Multiplemyeloma [151]
MK2206	Akt	Inhibit Survivin-XIAP complex	Glioblastoma [152]
SH5	Akt	Inhibit phosphatidyl inositol binding domain and Akti-X	Chronic myeloid leukaemia, lung and Prostate [153, 154]
TG101209	JAK2	–	CML, Lung [163, 164]
Gefitinib	EGFR	Dephosphorylation of EGFR	Lung, Breast [157]
Lapatinib	EGFR and HER-2	Upregulation of BMI and attenuation of ErbB1 and ErbB2 phosphorylation	Pancreatic, Breast and Ovarian [155, 156]
Trastuzumab	ErbB2	Transcriptional inactivation thorough β-Catenin/TCF	Breast [214]
Wnt-2Ab	Wnt		Lung [215]
PD153035	MAPK/ EGFR	H4 acetylation via TSA activation	Breast, Ovarian, Pancreatic, Lung, Colorectal and Blood [158–160]
AG1478			
PD98059			
CI1040	MEK	Blocking phosphorylation of ERK	Leukaemia, Breast, Keratinocytes [161]
U0126			
Bay-11-7057	IKK	Degradation of IĸB and inhibition of NF-ĸB	Pancreatic and Endothelial [182]
SD1029	JAK2	Combined inhibition of IAPs	Breast, Ovarian [168]
Panepoxyd- one	NF-ĸB	Regulation of IAPs	Breast [162]
Limonoids		Increase caspase 2 activity	Cervical [172]
Arctigenin	Stat3	Phosphorylation of Stat3,	NSCLC, Ovarian [165, 166]
Resveratrol		–	Cervical [169]
Tambjamine	JAK2 and IL-6	Stat3 phosphorylation	Lung [167]
GDP366	Survivin	Chromosomal Instability, Senescence	Leukaemia, Colon, Cervical [183]
Curcumin	NF-ĸB, Stat3, Akt	Multiple mechanisms	Breast, Ovarian, Liver cholangiocarcinoma [170, 243]
Etodolac Celecoxib	Cox2	PI3K/Akt suppression	Glioblastoma, Colon, Liver [173–175]
AKAP145	TGF-β	Dissociates Survivin/XIAP complex	Colon [184]
Metformin Phenformin		Affects Stat3, TGF-β and SMAD	Rectal [185, 226]
Salinomycin		Survivin downregulation	Renal cancer and CSCs [222]
IGC-001	β-catenin	Transcription inhibition and caspase activation	Colon [179], CSCs [237–239]
Emodin		Wnt /β-Catenin signalling regulation	Pancreatic [176, 177]
WM-127	Wnt	Supress β-catenin /TCF signalling induces Bax expression	Liver [178]
Xanthohumol Bulbine Futescens	Notch	Survivin suppression and affects stemness	Breast, Liver, Ovarian [180, 181]
SNS-032	CDK7/9	Downregulation of Survivin	Uveal melanoma [219]
Sabutoclax,	IL-6/Stat3		Breast CSCs [229]
CEP-1347	P38/MAPK		Ovarian CSCs [234]
Brexpiprazol	–	Reduction of Survivin	Pancreas, Lung, Brain CSCs [240]
AS602801	–	Combined downregulation of survivin and MDR1	Ovarian CSCs [248, 249]
Cardamonin	Stat 3	Survivin downregulation	GSCs [244]
Apigenin	PI3K/Akt	p53 and Apaf1 upregulation	Pancreatic CSCs[242]
PD173074	FGFR1	FGFR1/Scr/NF-ĸB axis	Pancreatic ductal adenocarcinoma CSCs [228]
mRNA Inhibitors			
LY2181301 (AO)	3′ untranslated region	Target mRNA cleaving, caspase activation	Lymphoma, Leukaemia, Melanoma, Breast [187]
SPC3042 (AO)	Exon 4	Target mRNA cleaving	Prostate, Lung [187, 189]
Oligo4003 (AO)	232–251 nucleotides	Nuclear condensation, Apoptosome formation	Lung [190]
siRNA (RNAi)	Binding to RISC to Target RNA	Target RNA cleaving	Pancreatic, Breast, Colorectal, Glioblastomas [183, 233, 234]
miRNA (RNAi)	3’-UTR	Target RNA cleaving	Prostate, Pancreatic, Lung, Gastric, Breast, Liver, Bladder, CSCs [194–198, 253–256]
Ribozymes	GUC(294) or CUA(110) of 3’ end	Hydrolysis of phosphodiester bond	Melanoma, Prostate [201, 202]

### Small Molecule Inhibitors

#### Inhibitors of Survivin Transcription

FL118, inhibits survivin and shows anti-tumor activity in p53 independent manner even at very low doses. It is known to possess excellent anti-tumor activity against colon, prostate, head and neck, ovarian and pancreatic cancers and in multiple myeloma in comparison to the already existing chemotherapeutics [116, 117]. It is also capable of selectively and independently inhibiting other anti-apoptotic genes including Mcl-1, XIAP and cIAP2 and has a favorable toxicity profile [118]. YM155 was identified as the first specific inhibitor of survivin that suppressed the activity of survivin promotor and showed promising results in human cancer cell lines and human hormone-refractory prostate tumor xenografts [119]. Even in phase I trials it was well tolerated and was effective against blood cancers to some extent. Surprisingly, in phase II trials on patients with refractory and advanced non-small-cell-lung-carcinoma (NSCLC) it was only as effective as other second-line agents [120]. It turned out that the suppression of survivin by YM155 was a secondary event and primarily it acts as a DNA damaging agent. Survivin repression was probably a result of reduced transcription [121].

Tolfenamic acid (TA) belonging to Nonsteroidal anti-inflammatory drugs **(**NSAIDs) causes post translational attenuation of SP1 and Sp3, which in turn results in the downregulation of survivin. TA in combination with Cisplatin has also been shown to reduce survivin levels in pancreatic and ovarian cancers [122, 123]. Copper II loaded complex of TA was found to be even more efficient in reducing survivin levels in pancreatic cancers[123]. Plant based Terameprocol which is in phase II studies, inhibits survivin expression in combination with cdc2 in cervical intraepithelial neoplasia. Their use has also shown positive effects on NSCLC, breast, colorectal, prostate, liver and erythroleukemia [124, 125]. Anti-neoplastic antibiotic Mithramycin A induces a similar effect and reduces cell viability in Ewing sarcoma and colon cancer [126, 127].

#### Inhibitors of Protein-Protein Interaction

Abbott-8, a set of small molecules developed computationally and identified using NMR, binds to survivin dimer interface inhibiting the symmetric interaction between two Survivin molecules. Inhibition of dimerization results in the exposure of hydrophobic interface of the protein resulting in conformational changes, causing selective degradation of survivin [79, 128]. *In silco* screening for compounds targeting the critical core residues for Survivin led to the identification of LQZ7 and its analogue LQZ7F, both of which could induce proteasome dependent degradation of Survivin. LQZ7F is less cytotoxic in comparison and has the potential to cause mitotic arrest, induce apoptosis and also inhibit tumor growth in mice [129].

Some compounds bind to survivin at Smac binding region, preventing the binding of Smac/DIABLO, making it available for inhibiting IAPs. Piperine, an alkaloid isolated from black pepper, works by this mechanism in colon and breast cancer and also in breast stem cells increasing bioavailability of drugs and decreasing drug metabolism [130, 131]. Leaf extract of Ashwagandha plant, Withanone, mimics Smac and inhibits survivin in cancers [132]. UC112 and its analogues 4 g and 10f, also compete with Smac to selectively downregulate Survivin through ubiquitin mediated pathway in melanoma, pancreatic and prostate cancers with low toxicity [133].

The disruption of the interaction between survivin and heat shock protein 90 (Hsp-90) destabilizes survivin, lowers antiapoptotic threshold and suppresses cell proliferation [134, 135]. Shepherdin, a peptidomimetic antagonist of Hsp90, leads to proteasomal degradation of survivin [136, 137]. AICAR destabilizes Survivin along with other HSP90 client proteins in tumors [133, 138]. A geldanamycin, 17AAG and a non-geldanamycin NYP-922 are two other Hsp90 inhibitors found to regulate survivin in myeloid leukemia, thyroid, lung, nasopharyngeal, cervical and colorectal cancers [139, 140].

#### Inhibitors of Mitosis

The in vitro studies on breast cancer cell lines showed the ability of an HIV treatment molecule Indinavir to cause mitotic arrest and apoptosis. They act by inhibiting chromosomal passenger complex (CPC) formation by phosphorylation of Aurora B kinase and by inhibiting survivin-XIAP binding. Another molecule S12 binds to survivin altering microtubule dynamics by specifically targeting mitotic check point and leads to cell death without being cytotoxic [141]. Computationally developed LLP3, disrupts the formation of Survivin-Ran complex, affecting microtubule stability and spindle formation in tumors [64].

Cyclin dependent kinase inhibitors like flavopiridol or purvalanol act by causing hypophosphorylation of survivin resulting in the loss of its function. This leads to inhibition of taxol-mediated mitotic block, activation of intrinsic and p53 independent apoptosis. Flavopiridol is in phase II clinical trial [142]. Sequence specific inhibition of CDK-1 with Taxol-Purvalanol A showed sequence and drug dependent pro-apoptotic efficacy that resulted from reduction in survivin expression [143]. A novel inhibitor Nu6140 suppresses survivin expression resulting in cell cycle arrest and increased apoptosis [144]. Cephalochromin, an isolate obtained from fermented fungi, has the potential to downregulate survivin, disrupt mitochondria and bring about cell cycle arrest in lung cancer cells [145].

#### Inhibitors Targeting Signaling Pathways

A secondary metabolite Prodigiosin and Omega-3 fatty acids (docosahexaenoic and eicosapentaenoic acid), downregulate survivin expression through p53 dependent pathways in ALL, AML, breast, liver and colorectal cancers [146–148]. MDM2 inhibitors, Nutlin 3a and RG7388, alone and in combination, suppress survivin at mRNA and protein levels, by activation of p53 and inhibition of mTOR pathways in neuroblastoma [149]. Inhibition of integrin-α5β1 by Nutlin 3a also results in induction of apoptosis in glioblastoma cell lines due to the proportional relation between survivin and integrin-α5 [150]. Rapamycin, a macrolide antibiotic, indirectly reduces survivin by inhibiting mTOR in glioblastoma, leukemia, lymphoma and multiple myeloma [151].

An allosteric kinase inhibitor of Akt, MK-2206 induces cell cycle arrest and apoptosis by preventing survivin/XIAP complex formation [152]. Similarly Akt specific SH5, also reduces survivin expression along with the significant reduction in other IAPs in chronic myeloid leukemia (CML), lung and prostate cancer [153, 154]. A dual kinase inhibitor, Lapatinib inhibits survivin in pancreatic, breast and ovarian cancers through the down regulation of ErbB1 and ErbB2 phosphorylation or upregulation in BMI1 expression [133, 155, 156]. EGFR inhibitors (Gefitinib, PD153035 and AG1478), MAPK antagonist (PD98059) and PI3K inhibitor (LY294002) have shown their potential to inhibit survivin in breast, lung, pancreatic, colon and ovarian cancer cell lines [157–160]. Specific MEK inhibitors CI1040 and U0126 were found to attenuate survivin levels in leukemia, with considerable reduction in cell viability through MAPK/ERK and PI3K/Akt pathways [161].

Panepoxydone isolated from edible mushroom causes combined downregulation of many IAPs in breast cancer cells without any cytotoxicity [162]. JAK2 inhibitor TG101209, suppresses survivin expression at nanomolar concentrations increasing susceptibility to chemotherapy in CML and radiotherapy in lung cancer [163, 164]. Arctigenin, isolated from the seeds of burdock and indole based Tambjamine, prevents Stat3 phosphorylation [165–167]. SD-1029 inhibits anti-apoptotic proteins downstream of JAK2 and increases sensitivity to paclitaxel in breast and ovarian cancer [168]. Resveratrol downregulates survivin and induces TRAIL based therapy in cervical cancers [169].

Curcumin, a broad spectrum anticancer drug was found to downregulate NF-ĸB, Akt and Stat3 by directly or indirectly reducing levels of survivin, thus inducing both intrinsic and extrinsic apoptosis [170, 171]. The neem limonoids, azadirachtin and nimbolide, reduce survivin levels through NF-ĸB in human cervical cancers [170, 172]. Etodolac and celecoxib, that selectively inhibit COX-2, are found to bring about significant survivin reduction in gliomas, colon and liver cancers thus improving efficacy of chemotherapy drugs [173–175]. Plant based compounds like WM-127, physodic acid, caperatic acid and Emodin induce Wnt mediated survivin reduction in cancers of liver colon and pancreas [176–178]. IGC-001, a β-Cantenin/ T Cell Factor mediated transcription antagonist, downregulates survivin expression leading to selective caspase activation in tumor cells [179]. Silencing Notch, using peptide antibiotic echinomycin, natural phytochemicals Xanthohumol and Bulbine frutescens, silences survivin in breast, ovary, liver and cholangiocarcinoma and has significant effects on stemness [180, 181]. Other small molecules including Bay-11-7057, AKAP145, Metformin, Phenformin, GDP366, etc. have also been found to be useful survivin inhibitors [182–186].

### mRNA Inhibitors

#### Antisense Oligonucleotides (AO)

Ely Lilly & Co. produced the first AO, LY2181308, a second-generation oligonucleotide, that binds to 3’-UTR region of survivin transcript, leading to destruction of survivin RNA by RNAse H [187]. This AO has been discontinued after phase II trials due to disappointing results [188]. Another AO that has reached clinical trials is SPC3042 (EZN-3042), targeting exon 4 of the survivin transcript. They act by initiating cell cycle arrest at the short window between metaphase and anaphase and activating caspases 3 and 7 [187, 189]. Oligonucleotide 4003, targets the mRNA (232–251 nucleotides) of survivin. This results in nuclear condensation and DNA fragmentation along with caspase activation [190]. It was withdrawn after phase I trials due to intolerable dose-limiting toxicity levels when in combination with chemotherapeutic drugs [191].

#### RNA Interference (RNAi)

Silencing of *survivin* in human androgen independent prostate cancer using RNAi reduced the proliferative potential and elevated the rate of apoptosis [192]. siRNA directed against *survivin* in neuroblastoma cell lines completely suppressed the expression at both mRNA and protein levels [193]. Micro RNAs (miRNAs), including miR-203, miR-34a, miR-218, miR-138p, miR-485-p, miR-542-3p and miR-214-3p, delivered to malignant cells using viral or non-viral based delivery systems, bind to survivin and induce gene silencing. miR-203 is capable of direct targeting in prostate, laryngeal and pancreatic cancers and inhibiting proliferation [194, 195]. Overexpression of miR-34a in head and neck squamous cell carcinoma, NSCLC, laryngeal squamous cell carcinoma and gastric cancer caused survivin downregulation [196, 197]. miR-214-3p tetrahedral framework nucleic acids complex, reduces survivin expression in lung cancer inducing apoptosis [198]. Survivin repression by other miRNAs resulted in reduction in proliferation, invasion and reversal of chemosensitivity in cancers of bladder, liver and breast [199, 200].

### Ribozymes

Small RNA molecules with endonucleolytic activity that cleaves target RNA sequence by catalyzing hydrolysis of phosphodiester bonds are regarded as ribozymes. They can act as transcriptional inhibitors of survivin activity [190]. Studies on human melanoma cell lines transfected with hammerhead ribozymes like CUA110 (RZ7) and GUC294 (RZ1), result in reduction of survivin expression that contributes to cell death and sensitivity to drugs and radiation. Attenuation of survivin expression with ribozymes improves susceptibility of melanoma cells to anti-tumor activity of cisplatin and topotecan triggering apoptosis [201, 202].

### Immunotherapy

#### Survivin Derived Peptides

Survivin stimulates cytolytic T cell response, when corresponding peptides are loaded to Major Histocompatibility Complex (MHC). The MHC-peptide complex when presented to CD 4+ or CD8+ T cell through dendritic cells (DC) or other antigen presenting cells (APC), causes subsequent lysis of the human leukocyte class I or II antigen (HLA) matched target cancer cell [49, 203–205]. Autologous DC pulsed with survivin derived epitopes has been able to instigate CD4+ and CD8+ T-cell responses in patients with ALL reducing the chances of relapse [206]. Recombinant melanoma-associated antigen and recombinant survivin when used in combination was found to be effective for lung cancer [207]. Similarly survivin derived peptides were useful immunotherapeutic targets in prostate carcinoma, chronic lymphatic leukemia and malignant melanoma [208]. Adoptive T cell therapy that identifies survivin specific clone and discriminate cancerous and non-cancerous cell has been identified to be an effective therapeutic method [209]. Combination therapy using adoptive T cell transfer and tumor antigen vaccine, derived from peptides of human telomerase reverse transcriptase and survivin, after autologous stem cell transplantation, produced enhanced cellular and humoral host antitumor immunity [210].

#### Cancer Vaccines

The possibility of utilizing survivin based vaccines as a potential immunotherapeutic agent in colorectal, pancreatic and lung cancer, renal cell carcinoma and neuroblastoma has been reported [125, 211]. Survivin DPX-Survivac, a depot-based cancer vaccine is derived from survivin and contains multiple CD8 epitopes with significant HLA applicability. This vaccine in combination with metronomic cyclophosphamide in a low oral dose, was able to generate polyfunctional antigen specific immune response in ovarian cancer patients [212]. Subunit vaccine formulation containing survivin and costimulatory SA-4-1BBL has been found to eradicate lung carcinoma, with increased efficiency when given in a prime-boost dose. Combination of peptide vaccines of survivin and indoleamine 2,3-dioxygenase with temozolomide was able to initiate specific immunity in glioma patients [213]. Many of the survivin based vaccines have entered Phase I or II of clinical trials and are reported to be relatively safe, with improved specificity for tumor cells.

#### Monoclonal Antibodies

The potential of monoclonal antibodies to target survivin has also been reported in several cancers. They are HLA independent and have more immediate action in comparison to cancer vaccines [14]. Trastuzumab targets ErbB2 and prevents *survivin* transcription through the binding of β-Cantenin/T cell factor to survivin promotor in breast cancer [214]. You *et.al.* developed a monoclonal antibody Wnt-2 Ab against Wnt protein that attenuates survivin through the suppression of the same pathway in lung cancers [215].

## Therapeutic Strategies Targeting Survivin in CSCs

A multitude of strategies have been developed and explored to target CSCs from bulk tumors. Targeting the factors helping in self-renewal, pathways promoting stemness in CSCs and pathways helping in CSC proliferation are approaches capable of reducing the frequency of CSCs considerably [216, 217]. A form of immunotherapy called oncolytic virotherapy, using competent replicating viruses, kills CSCs by hijacking the cell death machinery, inducing anti-tumor immunity, destroying tumor vasculature or using their toxic proteins to kill the cells directly [218]. Use of mRNA inhibitors and targeted drug delivery using nanoparticles are also extremely promising approaches. The most well established one is combination therapy utilizing the synergistic effect of survivin inhibitor and standard treatment modalities in eliminating these cells. Other modalities including the targeting of DNA repair pathways, CSC niche and differentiation therapy have also been explored [216].

### Targeting Factors Promoting Stemness and Key Signaling Pathways

Treatment with SNS-032, a selective inhibitor of CDK7/9 showed dramatic decrease in the IAP levels in uveal melanoma and subsequent elimination of CSCs and reduction in liver metastasis [219]. TmSm (T34A), a recombinant protein targeting survivin had prominent effect reducing cell growth and proliferation and inducing apoptosis in breast CSCs. The protein downregulates Cyclin D1 and Rb, and interferes with survivin/β-catenin interaction [220]. Mitotic inhibitor LLP3 was found to be useful in targeting survivin in GSCs by decreasing their proliferation [64]. Aspirin was found to reduce proliferation and invasion in GSCs with downregulation of survivin along with Notch 1, Sox 2 and Stat 3 [221].

Salinomycin downregulates survivin through TGF-β signaling in renal cell carcinomas and at the same time was found to reduce stemness in the CSCs [222]. Aripiprazole, an antipsychotic with excellent safety profile, inhibits survivin probably through Wnt/β-Catenin mediated pathway in CSCs, sensitizing them to standard chemotherapeutic drugs. Similar effect is also produced by another antipsychotic drug, Olanzapine [223, 224]. Mithramycin A was found to be effective in reducing stemness of LSCs with the help of SP1 and c-Myc with significant downregulation of survivin expression as well [225]. Low concentration of Metformin inhibits EMT and chemosensitizes CSCs of breast, pancreas and ovary by downregulating the IAP [226]. Inhibitor of nuclear factor kappa-B kinase subunit beta (IKK β) inhibitor Cmpd4 inhibits IKK β/NF-ĸB signaling pathway with downregulation of survivin, along with stem cell and EMT markers in prostate CSCs [227]. Fibroblast Growth Factor Receptor (FGFR) 1 inhibitor PD173074 suppresses the FGFR1/Scr/NF-ĸB signaling in pancreatic ductal adenocarcinoma CSCs with significant reduction in expression of stem cell markers as well as IAPs [228].

An antagonist of Bcl-2 family proteins, Sabutoclax, downregulates survivin through IL-6/Stat3 signaling pathway, to improve chemosensitivity in breast cancer CSCs [229]. Disulfiram, a drug capable of de-regulating various factors associated with tumorigenesis, induced apoptosis via caspase 3 activation in presence of copper in triple negative breast CSCs. This drug downregulated Stat 3 signaling with subsequent effects of survivin and cyclin D1 leading to CSC elimination [230]. n-3 Polyunsaturated fatty acids induce growth inhibition in drug resistant CR-CSC through survivin downregulation and caspase-3 activation [231]. p53 activators, Prodigiosin and Omega-3 fatty acids were found to be useful survivin suppressors in breast and colon cancer CSCs [147, 232].

Synergistic effect of growth factor Midkine and natural flavonoid quercetin was able to bring about cell cycle arrest and apoptosis in prostate CSCs by downregulating survivin along with p38, ABCG2 and NF-ĸB via PI3K/Akt and MAPK/ERK pathways [233]. A small molecule kinase inhibitor CEP-1347, which has proven anti-tumor and more specifically anti-CSC activity, chemo-sensitizes ovarian CSCSs by downregulation of survivin through c-Jun N-terminal kinase (JNK) and p38/MAPK pathway [234]. FL118 has been used to reduce migration and invasion in NSCLC and breast cancer derived CSCs by downregulating survivin expression and drug resistance inducing proteins, thus increasing the sensitivity to chemotherapeutic treatments [235, 236].

### Combination of Small Molecule Inhibitors with Standard Chemo or Radiotherapy

Combination of ICG-001 with standard chemotherapy eliminates CSCs in leukemia, salivary gland squamous cell carcinoma and pancreatic cancers. This Wnt/β-catenin modulator induces forced symmetric differentiation to safely eliminate drug resistant CSCs [237–239]. Synergistic effect of FH535 and sorafenib attenuated survivin levels that subsequently brought about inhibition of liver CSCs via Wnt/β-catenin pathway inhibition [114]. Brexpiprazol, a serotonin-dopamine activity modulator reduces survivin expression along with significant reduction in Sox-2 and sensitizes CSCs of lung, pancreas and brain to chemotherapy [240, 241].

A natural flavonoid apigenin increases sensitivity to cisplatin in prostate CSCs. Its combination with cisplatin decreases the migratory and proliferative potential via PI3K/Akt pathway and induces apoptosis by upregulating caspase-8, Apaf-1 and p53 [242]. Curcumin in low doses upregulates MAPK pathway, downregulate Stat3 signaling and inhibit self-renewal and survival in GSCs in reactive oxygen species dependent mechanism [243]. Cardamonin, a plant based dietary compound, has significant effect on downregulation of Stat3 signaling followed by subsequent downregulation of survivin in CD133^+^ GSCs, increasing their sensitivity to temozolomide [244]. Bortezomib also increases the sensitivity of GSC’s to temozolomide in a dose and time-dependent manner by downregulating the FOXM1/survivin signaling pathway [245]. Diuretic, spironolactone sensitizes CSCs to Gemcitabine and Osimertinib [246]. Rho-assisted protein kinase inhibitors sensitize pancreatic CSCs to Gemcitabine by downregulating survivin [247].

AS602801 sensitizes ovarian CSCs to carboplatin and paclitaxel through combined reduction in survivin and multi drug resistant protein 1 (MDR 1). This is a candidate drug for targeting tumor initiation and invasion in CSCs [248, 249]. Synergistic effect of suberoylanilide hydroxamic acid with Imatinib in LSC, downregulates co-expressing Bcr/Abl, Survivin, MDR-1 and histone deacetylases, reduces resistance to Imatinib and induces cell death signaling [250].

### RNAi Based Treatment Modalities

Aptamer-siRNA chimera has been used to suppress survivin expression in doxorubicin resistant breast CSCs and this combination was capable of targeting both CSCs and the bulk cancer cell population [251]. Elimination of CSCs in CR-CSCs was achieved by aptamer mediated delivery of survivin siRNA in combination with chemotherapeutic agent 5-fluorouracil which otherwise is ineffective in targeting CSCs [252].

miR-136 was found to downregulate survivin in Notch dependent pathway inhibiting cell migration, angiogenesis and chemoresistance in ovarian CSCs and enhance the chemosensitivity to paclitaxel [253]. Survivin is a direct target of miR-203 and an indirect target of miR-29b, hence miR-203/survivin/Bmi1 and miR-29b/SP1/fucosyltransferase 4 axes are useful in targeting LSCs [254, 255]. Downregulation of c-Myc, Survivin and β-Catenin by miR-147 leads to inhibition of EMT and subsequent loss of stem like traits in colon CSCs [256].

### Oncolytic Virotherapy

Oncolytic Virotherapy using survivin responsive conditionally replicating adenoviruses (CRAs) regulated with multiple tumor specific is another effective approach with proven ability to target chemoresistant CSCs and tumorigenic pluripotent stem cells. These viruses specifically replicate in and induce cell death in cancers and CSCs. The high survivin promoter activity, improved selectivity and specificity of survivin responsive CRAs, in comparison with other CRAs, make them more effective in treatment, alone or in synergy with radio or chemotherapy [257]. Survivin utilizing CRAs, that bind to heparan sulfate proteoglycans, improve sensitivity to radiotherapy and specifically target CD133^+^ GSCs [258]. They effectively target FGFR3 positive Rhabdomyosarcoma CSCs as well [259]. In an attempt to improve the efficiency of delivery of Survivin responsive CRAs, neuronal stem cells were used as carriers. This was effective in targeting ovarian CSCs in combination with cisplatin with minimal immunogenicity and clinical safety [260]. Many of these CRAs are under clinical trials.

### Targeted Drug Delivery Using Nanocomplexes

Nanocomplexes with increased bioavailability, biocompatibility, sustained and controlled drug releasing potential and high efficacy are promising targeted drug delivery systems. Considerable downregulation in survivin expression was obtained using iron-saturated bovine lactoferrin nanocarriers in colorectal CSCs [261]. Specific drug delivery to target GSCs has been accomplished with a cationic liposome nanocomplex delivering a combination of survivin siRNA and Paclitaxel with relatively less toxicity [262]. Co-delivery NF-ĸB inhibitor, IMD-0354 and chemotherapeutic agent Doxorubicin, encapsulated in ligand targeted nanoparticle, effectively targeted tumor microenvironment by increasing sensitivity and reducing cytotoxicity of Doxorubicin [263]. Chitosan nanoparticle encapsulated dominant negative survivin, competitively inhibited the endogenous protein, specifically in survivin overexpressing CSCs [264]. They have been used in delivery of survivin inhibitors, alone or in combination with chemotherapeutic agents for synergistic downregulation of survivin. They have also been used in survivin based gene therapy. Drug delivery using these nanocomplexes have been reported in cancers of breast, brain, lung, ovary and prostate [265, 266].

## Conclusions

Survivin will be an indispensable molecule in cancer therapeutics due to its differential expression in almost every cancer and its near absence from adult differentiated normal tissues. Despite survivin having this attractive distinction and strong preclinical data there has been only a partial success with anti-survivin treatments.

One of the possible reasons could be the lack of true survivin-specific inhibitors as observed in the case of small molecule inhibitor YM155 and AO LY2181308. Small molecules are required to disrupt the protein-protein interactions between survivin and other proteins. This entails significant challenges as most interfaces in such protein-protein interactions do not show effective small molecule binding [267]. This has resulted in the development of only a few survivin-specific inhibitors whereas majority of the inhibitors reduce survivin expression by indirectly acting on survivin. These indirect inhibitors show their effect by the combination of survivin inhibition along with other mechanisms of action [157, 268].

In case of AO therapy, an important factor impacting its efficacy is the target specificity which is difficult to attain. This is because RNA exists as a three-dimensional structure and the efficacy of an AO depends on the extent to which it is able to bind to the exposed regions of the RNA.

The limited success with these inhibitors may also be due to tumor heterogeneity. Lack of suitable assays for screening various drug candidates is also a hindrance to the development of clinically relevant small-molecule survivin inhibitors. Survivin being a nodal protein interferes with numerous processes like autophagy, mitosis and apoptosis and with a range of molecules like caspases, Hsp90, Smac/DIABLO etc. This makes it difficult to measure the target specificity of any drug [269]. Overcoming this would require optimized assay development.

Overall the current major challenge would be to design strategies that directly and precisely target survivin. This may be possible with homo-dimerization inhibitors or with immunotherapeutic methods. Nanocomplexes would be very promising for the delivery of such anti-survivin therapeutics. Another important avenue that needs to be explored is the possibility of combinatorial approach which would include survivin inhibitors along with current standard treatment modalities for synergistic effect. Molecules capable of targeting stemness promoting factors and the related signaling pathways are very promising. Combining RNAi or immunotherapeutic methods with conventional therapy also seems reassuring. Collectively, survivin inhibition in CSCs with the current standard treatment regimens hold the maximum possibility.

## Abbreviations

IAP: Inhibitor of Apoptosis Protein
BIRC5: Baculoviral IAP Repeat (BIR) - Containing 5
INCEP: Inner Centromere Protein
HSCs: Hematopoietic Stem Cells
HSPCs: Hematopoietic Stem and Progenitor Cells
MSCs: Mesenchymal Stem Cells
ASCs: Adipocyte-Derived Stem Cells
ES: Embryonic Stem Cells
HES: Human Embryonic Stem Cells
CSCs: Cancer Stem Cells
CCCs: Circulating Cancer Cells
NSCLC: Non-Small Cell Lung Cancer
GSC: Glioblastoma Stem Cells
CR-CSCs: Colorectal CSCs
ALL: Acute Lymphoblastic Leukemia
AML: Acute Myeloid Leukemia
CLL: Chronic Lymphoblastic Leukemia
CML: Chronic Myeloid Leukemia
LSCs: Leukemia Stem Cells
SP1: Specificity Protein 1
CPC: Chromosomal Passenger Complex
XIAP: X Linked IAP
Smac/DIABLO: Second Mitochondria-Derived Activator of Caspase/Direct Inhibitor of Apoptosis Binding Protein With Low pI
HSP: Heat Shock Protein
VEGF: Vascular Endothelial Growth Factor
CDE/CHE: Cell Cycle-Dependent Element/ Cell Cycle Genes Homology Region
PI3K: Phosphoinositide-3 Kinase
FOXO: Forkhead Box O
PTEN: Phosphatase and Tensin Homolog
Ang-1: Angiopoietin-1
NF-ĸB: Nuclear Factor Kappa-Light-Chain-Enhancer of Activated B Cells
COX-2: Cyclooxygenase-2
GM-CSFR: Granulocyte-Macrophage Colony-Stimulating Factor Receptor
EGFR: Epidermal Growth Factor Receptor
HER: Human Epidermal Growth Factor Receptor
IGF: Insulin-like Growth Factor
mTOR: Mammalian Target of Rapamycin
CDK: Cyclin Dependent Kinases
Runx2: Runt-Related Transcription Factor 2
MDM2: Mouse Double Minute 2
Rb: Retinoblastoma Gene
GSK-3: Glycogen Synthase Kinase-3
HIF: Hypoxia Inducible Factor
JAK: Janus Kinase
Stat: Signal Transducer and Activator of Transcription
MAPK: Mitogen-Activated Protein Kinase
ERK: Extracellular-Signal-Regulated Kinase.
RAS: Retrovirus-Associated DNA Sequences.
RAF: Rapidly Accelerated Fibrosarcoma.
TGF-Β: Transforming Growth Factor Beta.
CXCR4: C-X-C Motif Chemokine Receptor 4.
SDF-1: Stromal Cell-Derived Factor.
Sox-2: SRY (Sex Determining Region Y)-Box 2.
Oct-4: Octamer-Binding Transcription Factor 4.
SHh: Sonic Hedgehog.
ITD-Flt3: Internal Tandem Duplication-Flt3 Tyrosine Kinase.
TRPM7: Transient Receptor Potential Cation Channel, Subfamily M, Member 7.
IKKβ: Inhibitor of Nuclear Factor Kappa-B Kinase Subunit Beta.
IL: Interleukin.
HIV: Human Immunodeficiency Viruses.
NMR: Nuclear Magnetic Resonance.
Nsaids: Nonsteroidal Anti-Inflammatory Drugs.
FGFR: Fibroblast Growth Factor Receptor.
MDR1: Multi Drug Resistant Protein 1.
miRNA: Micro Ribonucleic Acid.
siRNA: Small Interfering Ribonucleic Acid.
MHC: Major Histocompatibility Complex.
APC: Antigen-Presenting Cell.
DC: Dendritic Cells.
HLA: Human Leukocyte Antigen.
TCF: T Cell Factor.
TA: Tolfenamic Acid.
